# 3D Auxetic Metamaterials with Elastically‐Stable Continuous Phase Transition

**DOI:** 10.1002/advs.202204721

**Published:** 2022-10-18

**Authors:** Lianchao Wang, Gwenn Ulliac, Bing Wang, Julio A. Iglesias Martínez, Krzysztof K. Dudek, Vincent Laude, Muamer Kadic

**Affiliations:** ^1^ National Key Laboratory of Science and Technology on Advanced Composites in Special Environments Harbin Institute of Technology Harbin 150001 P. R. China; ^2^ Institut FEMTO‐ST CNRS UMR 6174, University Bourgogne Franche‐Comté Besançon 25000 France; ^3^ Institute of Physics University of Zielona Gora ul. Szafrana 4a Zielona Gora 65‐069 Poland

**Keywords:** 3D metamaterials, auxetic metamaterials, continuous phase transition, elastically‐stable, negative poisson's ratio

## Abstract

In solid state physics, phase transitions can influence material functionality and alter their properties. In mechanical metamaterials, structural‐phase transitions can be achieved through instability or buckling of certain structural elements. However, these fast transitions in one mechanical parameter typically affect significantly the remaining parameters, hence, limiting their applications. Here, this limitation is addressed by designing a novel 3D mechanical metamaterial that is capable of undergoing a phase transition from positive to negative Poisson's ratio under compression, without significant degradation of Young's modulus (i.e. the phase transition is elastically‐stable). The metamaterial is fabricated by two‐photon lithography at the micro‐scale and its mechanical behavior is assessed experimentally. For another choice of structural parameters, it is then shown that the auxetic behavior of the considered 3D metamaterial class can be maintained over a wide range of applied compressive strain.

## Graphical abstract

In this work, a novel 3D mechanical metamaterial that is capable of undergoing a phase transition from positive to negative Poisson's ratio under compression is designed, without significant degradation of Young's modulus (i.e. the phase transition is elastically‐stable). Moreover, the auxetic behavior of the considered 3D metamaterial can be maintained over a wide range of applied compressive strain.

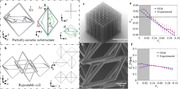

## 1 Introduction

Rationally designed artificial structural materials can have counterintuitive mechanical properties that are difficult to observe in natural materials.^[^ ^1^ ^]^ Structures capable of exhibiting such behavior are called mechanical metamaterials.^[^ ^2^ ^]^ In the past twenty years, a large number of mechanical metamaterials^[^ ^3^, ^4^, ^5^, ^6^, ^7^, ^8^, ^9^, ^10^, ^11^, ^12^, ^13^, ^14^, ^15^, ^16^, ^17^, ^18^, ^19^, ^20^ ^]^ have been designed and fabricated, thanks to the rapid development of 3D printing technology.^[^ ^21^, ^22^, ^23^, ^24^, ^25^, ^26^, ^27^, ^28^ ^]^ Some of the most appealing directions of research are related with the propensity of mechanical metamaterials to exhibit highly unusual mechanical behaviors such as auxeticity,^[^ ^7^, ^29^ ^]^ negative stiffness,^[^ ^30^, ^31^, ^32^ ^]^ multistable (programmable) behavior,^[^ ^8^, ^33^, ^34^ ^]^ acoustic insulation,^[^ ^5^, ^35^ ^]^ ultra‐high strength,^[^ ^36^ ^]^ ultra‐high stiffness,^[^ ^37^ ^]^ or doubly‐negative properties.^[^ ^6^ ^]^ Among these directions, auxetic structures have become one of the most commonly studied classes of mechanical metamaterials, as a result of their impressive compression‐shrinkage deformation characteristics.^[^ ^38^, ^39^, ^40^ ^]^ These special deformation modes enable auxetic structures to exhibit other forms of highly desired mechanical behaviors that are advantageous in comparison to conventional materials, e.g., remarkable shear modulus, large energy absorption capacity, indentation resistance, and adjustable porosity. Therefore, it is not surprising that auxetic metamaterials have been successfully applied in many engineering fields.^[^ ^39^, ^40^, ^41^ ^]^ Over the past four decades, negative Poisson's ratio mechanical metamaterials have been extensively studied.^[^ ^6^, ^7^, ^41^, ^42^, ^43^, ^44^, ^45^, ^46^, ^47^, ^48^, ^49^, ^50^, ^51^, ^52^, ^53^, ^54^, ^55^, ^56^ ^]^ Unfortunately, despite the large literature on auxetic mechanical metamaterials, very few papers report mechanical phase transitions.^[^ ^57^, ^58^, ^59^ ^]^

In chemistry, thermodynamics, and generally physical sciences, phase transitions are the physical processes of transition between states corresponding to different properties.^[^ ^60^ ^]^ They typically correspond to a change in the order of parameter that is defined depending on the investigated phenomenon and system. Furthermore, even though the concept of phase transition is typically used with an emphasis on magnetism, thermodynamics, etc., it can be also used in the case of mechanics to discuss the behavior of specific mechanical properties at different stages of a deformation process. In fact, over the years, it has been demonstrated that it is possible to observe various types of phase transitions in a variety of materials used in engineering. One of the most common example corresponds to phase transitions in shape memory alloys that can be exploited to introduce movement.^[^ ^61^, ^62^ ^]^ Moreover, for different mechanical systems, it was reported that phase transitions can affect the mechanical properties of systems^[^ ^63^, ^64^ ^]^ and further influence the conversion of energy.^[^ ^58^, ^65^, ^66^ ^]^

In the field of mechanical metamaterials, there are several examples where phase‐transitions were observed as a result of the application of external stimuli. For instance, in phase‐transforming structures with magnetic inclusions,^[^ ^58^ ^]^ it was shown that the resulting metamaterial can exhibit different static and dynamic mechanical properties, before and after being subjected to the external stimulus. Furthermore, in the case of nonmagnetic mechanical metamaterials, Farzaneh et al.^[^ ^57^ ^]^ studied sequential metamaterials with alternating Poisson's ratios under both compressive and tensile loads. Other examples include the work by Janbaz et al.^[^ ^59^ ^]^ where a strain‐induced phase transition of Poisson's ratio was observed for a system consisting of a combination of stiff and soft bi‐beams. 2D^[^ ^67^ ^]^ and 3D^[^ ^68^ ^]^ auxetic metamaterials with a phase transition induced by the elastic instability or the post‐buckling of the raw materials of structures were demonstrated. However, despite those progresses it is worth mentioning that those reconfigurable mechanical metamaterials normally share one limitation: as one of the investigated mechanical properties undergoes a transition, the other mechanical properties are also affected that can be particularly problematic if we try to change only specific properties of the system. Nevertheless, as discussed in this work, it is possible to identify mechanical metamaterials capable of overcoming this obstacle.

In light of the above, a novel micro‐scale 3D auxetic mechanical metamaterial undergoing a phase transition in the value of the Poisson's ratio during its deformation process is proposed. To fully assess its properties, the behavior of the system is investigated for two different configurations that lead to a very different mechanical profile. More specifically, it is demonstrated that for the first of the considered configurations, it is possible to observe the phase transition in the value of Poisson's ratio while maintaining the trend of the change in the Young's modulus throughout the deformation process. The latter is very significant since as discussed above, such characteristic is not commonly observed for other mechanical metamaterials that were reported to be able to undergo a phase transition in their mechanical properties. On the other hand, for the second configuration, there is no phase transition and the Poisson's ratio exhibits the same trend throughout the deformation. It is also important to emphasize the fact that both specimens were fabricated via 3D printing technology, and their mechanical properties were studied by analytically, experimentally, and with Finite Element Method (FEM) computations. Finally, it should be mentioned that despite other differences, all structures investigated in this work exhibit a high specific energy absorption.

## 2 Results and Discussion

**Figure 1:**
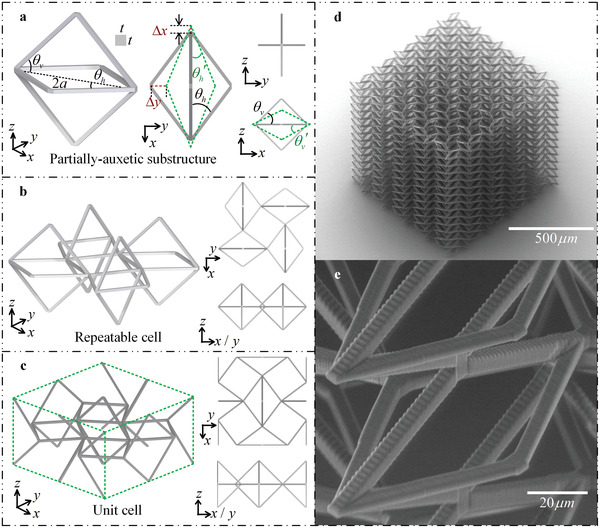
Design philosophy of the novel 3D auxetic metamaterial. a) The partially‐auxetic substructure has expansion behavior in the *x*‐direction and contraction behavior in the *y*‐direction when compressed in the *z*‐direction. For some specific parameters, contraction in the *y*‐direction is larger than expansion in the *x*‐direction, that is Δy > Δx. The green dotted line outlines the pre‐deformed shape in the xy‐plane when the substructure is compressed in the *z*‐direction. b) Repeatable cell obtained by alternately arranging the partially‐auxetic substructure along the x− and *y*‐directions. c) Unit cell with negative Poisson's ratio effect in all three principal directions. d) SEM images of the considered 3D mechanical metamaterial fabricated by two photon lithography with the custom IP‐S resin. e) A close‐up view taken from (d).

In this work, we consider a novel type of 3D partially‐auxetic substructure (see **Figure** **1**a) that can exhibit either the negative and positive Poisson's ratio depending on which deformation plane is considered. More specifically, if one was to stretch the considered substructure along the *z*‐axis, it would exhibit positive Poisson's ratio in the xz‐plane but auxetic behavior (negative Poisson's ratio) in the yz‐plane. From the geometry point of view, the *x*‐dimension of the undeformed partially‐auxetic substructure is denoted as 2*a* and the angles between the respective truss‐lattice beams, having a square‐like cross section corresponding to the linear dimension *t*, are denoted in Figure 1b as $\theta_{h}$ and $\theta_{v}$. At this point, it is important to emphasize that, as indicated the green dashed line in Figure 1b, for some specific parameters, as the system is being compressed along the *z*‐axis, contraction in the *y*‐direction is significantly larger than expansion in the *x*‐direction, i.e. Δy>Δx.

The repeatable structural element used in order to create the considered structure corresponds to a truss lattice consisting of four partially auxetic substructures that are connected to each other at their vertices, as Figure 1b shows. This specific design results in a novel 3D mechanical metamaterial exhibiting auxetic behavior in all three principal directions. However, it is important to note that the actual unit cell of the system, presented in Figure 1c, is a bit different than the aforementioned repeatable cell. The repeatable cell has a weaker boundary effect than the periodic unit cell when the number of elements is limited, so the former was used in experiments instead of latter. An example of the entire structure considered in this work is presented in Figure 1d and its details are demonstrated in Figure 1e.

In this paper, in order to assess the mechanical properties of the system, we primarily focus on two distinct instances of the considered mechanical metamaterial, referred to as model A ($\theta_{h}=46.4^{\circ }$ and $\theta_{v}=45^{\circ }$) and model B ($\theta_{h}=\theta_{v}=26.56^{\circ }$). The only difference between both models is the initial value of angles $\theta_{h}$ and $\theta_{v,}$ whereas all other geometric parameters (2a=120 µm, t=6 µm) are the same. The specific design parameters are reported in the Experimental Section.

### 2.1 Effective Elastic Properties

**Figure 2:**
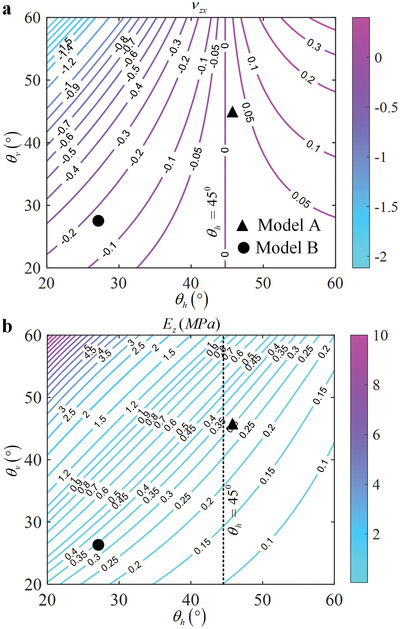
Variation of mechanical properties with structural parameters $\theta_{h}$ and $\theta_{v}$ in the range $\left[20^{\circ }-60^{\circ }\right]$. a) Poisson's Ratio and b) Young's modulus of the structure are plotted. It should be noted that the Young's modulus does not change significantly at the critical point (figured by the black dotted line in the figure) where the sign of Poission's ratio changes. Other geometric parameters are fixed for all configurations and are set to a=60 µm, t=6 µm, and E=4.5GPa.

In this work, to gain a better understanding of the fundamental mechanical properties of the considered system, its behavior is analyzed for different configurations of the structure corresponding to angles $\theta_{h}$ and $\theta_{v}$ assuming values in the range between 20° and 60°. At the same time, all other geometric values are kept constant and are set to a=60 µm, t=6 µm, and E=4.5GPa. Through the use of such parametric analysis, information is obtained about the mechanical performance for a broad set of possible geometric configurations. To this aim, two different mechanical properties are analyzed, i.e. Poisson's ratio and Young's modulus, in the case of the mechanical deformation corresponding to the infinitesimal strain applied along the *z*‐axis. Results from this analysis are generated by means of the analytical model that is presented in the Experimental Section. It is important to mention that the results were confirmed by means of computer simulations and experiments, with all approaches being in a good agreement as shown in (see Figure S1, Supporting Information). In addition, we emphasize that in a vast majority of known mechanical metamaterials, a change in one of the fundamental mechanical properties leads to a concurrent change in other mechanical properties. However, this is not the case in the present work as **Figure** **2** shows: the change of sign of Poisson's ration is not accompanied by a change in Young's modulus.

Regarding the results summarized in Figure 2, we emphasize the following points. The geometric parameters of the metamaterial have a significant effect on its equivalent elastic constants. However, the effective Poisson's ratio depends only on angles $\theta_{h}$ and $\theta_{v}$, while the equivalent elastic Young's modulus depends also on all other parameters of the structure. This fact illustrates the independence with size of the response of auxetic mechanical metamaterials. Moreover, when $\theta_{h}$ and $\theta_{v}$ vary in the range of $\left[20^{\circ }-60^{\circ }\right]$, the variations of Poisson's ratio are regular, i.e., the effective Poisson's ratio of the metamaterial increases with $\theta_{h}$ and increases or decreases with $\theta_{v}$. In practice, this effect is irregular or non‐linear. Here, we can consider two limiting cases, one when $\theta_{h}$ approaches 0 (in this case, the four beams in the *x‐y* plane tend to coincide and align parallel to the *x*‐axis), for which the partially auxetic structure has no deformability in the *y*‐direction, that is, Δy=0, as shown in Figure 1b. Poisson's ratio, in this case, is equal to zero. The second case is when $\theta_{h}$ is close to 90°, for which the strain of the partially auxetic structure in the *y*‐direction tends to 0, which leads to the strain of the metamaterial in the *x‐* and *y‐*directions to go to 0, i.e., Poisson's ratio is also 0. To sum up, the effect of the geometric parameters of the metamaterial on the effective Poisson's ratio is non‐monotonic. Furthermore, it is seen in Figure 2a that when $\theta_{h}<45^{\circ }$, the metamaterials are auxetic, that is, the critical condition for Δy>Δx in Figure 1b is that $\theta_{h}<45^{\circ }$. In contrast, the metamaterial has a positive Poisson's ratio when $\theta_{h}>45^{\circ }$. Although both $\theta_{h}$ and $\theta_{v}$ have obvious effects on the equivalent Poisson's ratio of the metamaterial, only $\theta_{h}$ has a decisive effect on its properties, that is, only $\theta_{h}$ affects whether the metamaterial has tension‐expansion or tension‐shrinkage behavior. Hence, the deformation characteristics of the metamaterial change drastically around $\theta_{h}=45^{\circ }$ (i.e. the sign of the Poisson's ratio flips), whereas the change in its elastic modulus remains smooth. In other words, the elastic modulus of the metamaterial does not change markedly with the variation of its deformation characteristics, at the mechanical phase transition.

At this point, we note that material post‐buckling or structural instability is a widely used mechanism for the design of metamaterials with phase transitions.^[^ ^67^, ^68^ ^]^ However, this mechanism has insurmountable drawbacks. Traditionally, both the instability of the structure and the buckling of the material indicate the failure of the system. As a result, the phase transition for metamaterials based on these mechanism is discontinuous and elastically unstable in the critical state.

In the remaining part of this work, the above results are used to examine under large mechanical deformation the two different configurations of the considered system referred to as model A and model B. Both structures correspond to different initial values of angles $\theta_{h}$ and $\theta_{v}$ that make it possible to explore the origin of the aforementioned changes in the value of Poisson's ratio.

### 2.2 Phase Transitions with Constant Young's Modulus and Sign Change of the Poisson's Ratio

**Figure 3:**
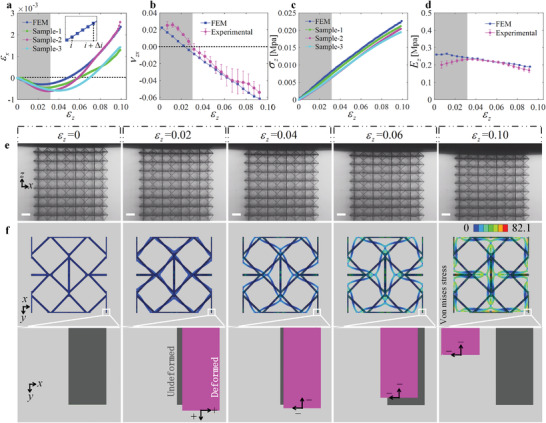
Mechanical properties of model A when it compressed in the z− direction. a) Normalized strain in the x−direction versus normalized strain in the z−direction. b) Tangent Poisson's ratio $\nu_{{}_{\mathrm{zx}}}$ versus normalized strain in the z−direction. c) Normalized stress versus normalized strain in the z−direction. d) Tangent Young's modulus versus normalized strain in the z−direction. In Figurea–d, the gray area represents the lateral expansion of the metamaterial, and the white area represents its lateral contraction. e) Experimental compression deformation; the scale bar length is 100 µm. f) Deformation process of the central unit cell obtained by finite element analysis. Displacements in the x− and the y−directions are magnified by a factor 8. Arrows indicate the direction of motion and the sign represents the positive or negative value of the tangent Poisson's ratio.

The main objective of this part is to evaluate the phase transition of the auxetic metamaterials. The mechanical response of experimental and simulation results of model A is shown in **Figure** **3**. Normalized strain in the z− direction versus normalized strain in the x−direction is plotted in Figure 3a. Positive values of $\varepsilon_{x}$ indicate shrinkage (gray area) of the metamaterial, whereas negative values indicate expansion (white area). In short, model A first expands and then contracts in the x−direction during the compression process, i.e. undergoing phase transition.

The effective Poisson's ratio is usually used to quantitatively evaluate the auxeticity of metamaterials. However, this quantity characterizes the deformation of the shape of the material relative to its original shape, hence it does not faithfully describe the deformation characteristics of the material, especially in the case of large deformations. For example, according to the definition of Poisson's ratio, the critical strain for the phase transition (compared to the initial shape, as shown in Figure 3a) of model A is ≈5%, that is, model A is a positive Poisson's ratio material before the compressive strain reaches 5%, and it becomes a negative Poisson's ratio material after the compressive strain exceeds 5%. In fact, the critical strain for the phase transition of model A is ≈3%, at which point the slope of the curve changes sign. As a result, we will consider instead the tangent Poisson's ratio to evaluate the deformation characteristics of auxetic metamaterials. This differential quantity is defined as the deformation size of the material at any step relative to the previous step. For both experiment and simulation, we obtain the value of the tangent Poisson's ratio via an approximation of the derivative of the compressive strain as a function of the transverse strain. Specifically, five data points between *i* and i+Δi are used to evaluate the tangent Poisson's ratio at the compressive strain of $i+\frac{1}{2}\Delta i$, as shown in the inset of Figure 3a. In this paper, the value of Δi is 0.5%.

The tangent Poisson's ratio for model A is shown as a function of the compressive strain in Figure 3b. Model A has a compressive‐expansion behavior when the compressive strain is <3%, but is auxetic when the compressive strain is >3%. The experimental and simulation results are shown in Figure 3e,f. Since the lateral deformation is relatively small, the deformation in both the x− and the y−directions is magnified in panel f by a factor 8. In the simulation results (shown by the magenta beams in Figure 3f), it can be seen that when the compressive strain is 0.02, model A exhibits an obviously positive Poisson's ratio effect. Moreover, it is still expanded in the transverse direction relative to the original shape (i.e. the compressive strain of 0%) when the compressive strain reaches 0.04, that is, positive Poisson's ratio behavior. However, relative to the shape at a compressive strain of 2%, it exhibits a negative tangent Poisson's ratio due to the absolute deformation of model A in both the x− and y−directions becoming smaller. This is because model A undergoes a phase transition when the compressive strain reaches 3%, i.e. from a positive tangent Poisson's ratio material to a negative tangent Poisson's ratio material, as shown in Figure 3b.

The strain‐stress and strain‐tangent modulus curves for model A are shown in Figure 3c,d, respectively. It is important to note here that the tangent modulus for model A does not change significantly during the entire compression process, and hence, at the phase transition, remaining within the range [0.18—0.28 MPa]. Since the simulation results do not take into account the elastoplasticity of the original material custom IP‐S resin, the slight change in the tangent modulus of model A is introduced by the geometric nonlinearity. The aforementioned properties are the primary beauty of the mechanical metamaterials defined in this work. In metamaterials based on curved beams,^[^ ^67^, ^68^ ^]^ the mechanical properties of auxetics decline sharply at the phase transition, since the latter is introduced by structural instability or material elastic buckling. In such case, although the phase transition broadens the functional applications of auxetics, the decrease in load‐carrying capacity limits the structural applications of auxetics. In contrast, thanks to the absence of instability or material buckling at the phase transition, the mechanical properties of model A do not decrease significantly although they drop slightly. It can be inferred that the value of $\theta_{h}$ decreases as the compressive strain increases. The phase transition occurs when the value of $\theta_{h}$ crosses 45°, at which the tangent Young's modulus does not change noticeably, as predicted by the model. It is concluded that the fundamental reason why model A has a phase transition is the geoetric adaptation of the metamaterial during the compression process, that is the compression changes the feature size and thus affects the deformation characteristics. The mechanical metamaterial undergoes a phase transition without losing its mechanical properties, which enables it to simultaneously meet the high requirements of modern industry for material multifunctional and structuring applications.

### 2.3 Large Strain Auxeticity

In principle, the characteristic properties of mechanical metamaterials mainly depend on the shape of their unit cell. This is the main advantage of mechanical metamaterials but at the same time a potential source of problem. Because the structure will eventually be destroyed at large deformations, auxeticity is only retained over a limited range of applied strain, especially for 3D auxetics. This disadvantage can limit the functional application of auxetics, for instance limiting their effective range as sensors. Therefore, this section mainly focuses on maintaining a regular negative Poisson's ratio under large compressive strain.

**Figure 4:**
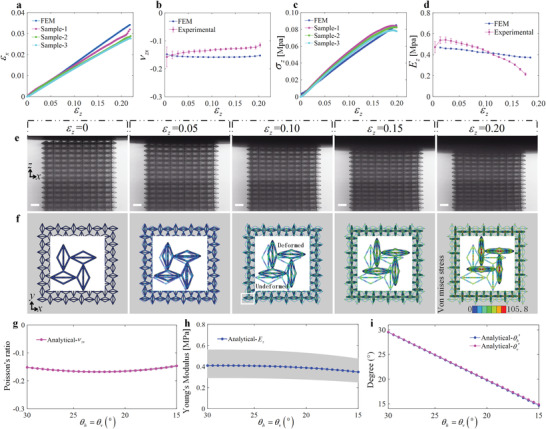
Mechanical properties of model B as a function of the applied compressive strain in the z−direction. a) Normalized strain in the x− direction versus normalized strain in z−direction. b) Tangent Poisson's ratio $\nu_{\mathrm{zx}}$ versus normalized strain in the z−direction. c) Normalized stress versus normalized strain in the z−direction. d) Tangent Young's modulus versus normalized strain in the z−direction. e) Experimental compression deformation process. The scale bar is 100 µm. f) Numerical simulation by finite element analysis of the deformation process of model B in the x,y plane. g–i) Theoretical variations of the equivalent Poisson's ratio, equivalent Young's modulus, and $\theta_{h}^{\prime }$, $\theta_{v}^{\prime }$ in the range $\left[15^{\circ }-30^{\circ }\right]$ for $\theta_{h}=\theta_{v}$. $\theta_{h}^{\prime }$ and $\theta_{v}^{\prime }$ represent the values of $\theta_{h}$ and $\theta_{v}$ after deformation. Other parameters are fixed as a=60 µm, t=6 µm, E=4.5GPa. Note that the upper and lower limits of the gray areas correspond to the cases of t=5.5 and t=6.5 µm, respectively.

The mechanical response of model B under larger compressive strain is summarized in **Figure** **4**. From Figure 4a,b, it can be seen that model B retains a regular negative Poisson's ratio for compressive strain in excess of 0.2. Simulations show that the tangent Poisson's ratio of model B is −0.16, and experiment yields a value of −0.14. The experimental results are obtained in the x,z plane and the simulation results are for the x,y plane during the compression process; they are shown in Figure 4e and f, respectively. Both results clearly show that model B has an almost constant negative Poisson's ratio for compressive strain up to 0.2, that is, Poisson's ratio of model B does not change with the deformation of the unit cell structure. This behavior is seldom seen in negative Poisson's ratio metamaterials, especially 3D auxetic metamaterials. The stress and tangent modulus curves versus compressive strain are shown in Figure 4c,d, respectively. The simulation results show that the tangent modulus of model B changes only slightly in the process of large deformation, and always remains ≈0.4MPa. However, although experimental and simulation results follow the same trends, experimental results show slight fluctuations, the reasons of which being explained later. It can be concluded that the equivalent elastic constants (Poisson's ratio and Young's modulus) of model B remain unchanged under large deformation. In other words, the mechanical properties of model B do not change with the deformation of its unit cell structure, i.e. the metamaterial retains an almost constant negative Poisson's ratio under a wide range of applied strain.

**Figure 5:**
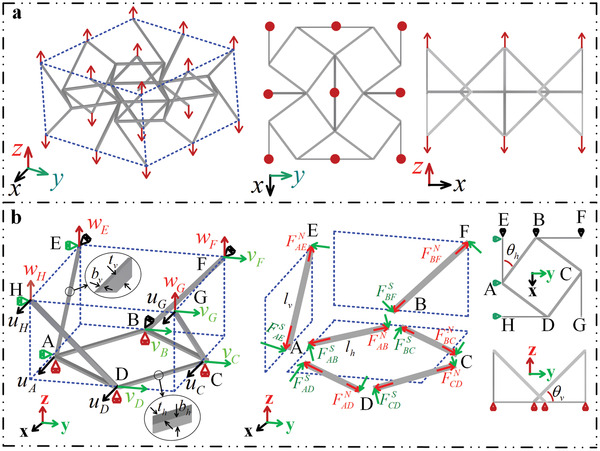
Force Analysis of representative volume elements of the metamaterial. a) The periodic unit cell subjected to tensile displacement in the *z* principal direction. b) Equivalent boundary conditions of a 1/8 unit cell under periodic boundary conditions, displacements and degrees of freedom of endpoints in three principal directions, and schematic diagrams of axial and shear forces for each individual beams. Note that the bending moments of each individual beams are not shown in this figure.

To reveal why the mechanical properties of model B do not change with the deformation of its unit cell structure, further theoretical calculations were performed. Figure 4g–i reports the theoretical variation of Poisson's ratio, Young's modulus, and the angles $\theta_{h}^{\prime }$ and $\theta_{v}^{\prime }$ in the range of $\left[15^{\circ }-20^{\circ }\right]$ for $\theta_{h}=\theta_{v}$. $\theta_{h}^{\prime }$ and $\theta_{v}^{\prime }$ represent the values of $\theta_{h}$ and $\theta_{v}$ after deformation, as defined in Figure 4b. It can be seen from Figure 4g,h that Poisson's ratio and Young's modulus of the metamaterial are almost constant when $\theta_{h}$ and $\theta_{v}$ are equal and in the range $\left[15^{\circ }-20^{\circ }\right]$. More importantly, the values of ${\theta_{h}}^{\prime }$ and ${\theta_{v}}^{\prime }$ also always remain equal under the above conditions, as shown in Figure 4i. Herein, the values after small deformation can be obtained by formulas $\theta_{h}^{\prime }=\arctan\left(\frac{a\tan\theta_{h}+v_{B}}{a+u_{A}}\right)$ and $\theta_{h}^{\prime }=\arctan\left(\frac{a\tan\theta_{v}+w_{E}}{a+u_{A}}\right)$, where $u_{A}$, $v_{B,}$ and $w_{E}$ are the displacements of points A, B, and E in three directions, respectively, as shown in **Figure** **5**. Here, $w_{E}=-1$ µm (corresponding to a compressive strain of 0.15%, with a negative sign indicating compression) to ensure that the deformation of the metamaterial remains small. The results in Figure 4g–i illustrate that under such conditions (i.e. $\theta_{h}$ and $\theta_{v}$ are equal and in the range $\left[15^{\circ }-30^{\circ }\right]$) the values of $\theta_{h}^{\prime }$ and $\theta_{v}^{\prime }$ always remain equal during compression, and Poisson's ratio and Young's modulus are constants. This is the primary reason that model B has a permanent negative Poisson's ratio effect under a wide range of applied strain. Furthermore, we consider changing the side length *t* of the beam section in the range [5.5–6.5 µm]. The equivalent elastic constants of the metamaterial is shown by the gray area in Figure 4g–i. It can be seen that only the Young's modulus of the metamaterial is sensitive to changes in *t*, but that the effective Poisson's ratio is rather insensitive. The fluctuations in the experimental results presented in Figure 4b,d are mainly caused by two factors. The first factor is error during sample preparation. The thickness and width of the beams of the sample are only 6 µm, but even a 0.5 µm error can cause a large change in Young's modulus, as shown in Figure 4h. The second factor is error during the compressive experiment. In the experiment, it is actually difficult to guarantee that the plate and the sample are absolutely vertical, that is, off‐axis compression may occur in the early stage of compression, which would further cause errors in the experimental results.

Moreover, another advantage of the considered metamaterial is that it has a large energy absorption capacity (see Section 2 of the Supporting Information). It can be seen that models A and B have different specific energy absorptions, 0.47 and 1.5 kJ kg^−1^, respectively. More importantly, models A and B have relatively low densities, 0.0138 and 0.0441 g cm^−3^, respectively. For such low‐densities, the auxetic metamaterial exhibits good energy absorption capacity, which is introduced for two main reasons. On the one hand, the raw material of the metamaterial, i.e. custom IP‐S resin, has outstanding specific mechanical properties (high elastic modulus and lower density). On the other hand, the studied metamaterial possesses structural efficiency due to the tension/compression‐dominated deformation mode of the metamaterial, while the counterpart of most other auxetics is bending‐dominated. Above all, the low density (light mass), the regular failure mode, the high specific energy absorption, and the energy absorption efficiency indicate that the proposed auxetic metamaterials have potential applications in the field of energy absorption, especially for advanced sport equipment and aerospace applications.

## 3 Conclusion

In summary, we have designed a novel 3D negative Poisson's ratio mechanical metamaterial that is elastically stable under large compressive deformations. By changing the feature size of the auxetic metamaterial, two different functional metamaterials are proposed. The first metamaterial displays a phase transition for Poisson's ratio that does not sacrifice the mechanical properties during the phase transition. The second metamaterial retains its auxetic behavior regularly over a wide range of applied strain. In addition, both metamaterials have high specific energy absorption and energy absorption efficiency. These superior properties make the auxetic metamaterials potential candidates for both functional and structural applications.

## 4 Experimental Section

### Fabrication

The micro‐scale samples studied in this paper were fabricated by 3D Direct Laser Writing. For this purpose, a commercial the two‐photon lithography method was used. The photoresist selected to produce the analyzed auxetic mechanical metamaterials was the commercial negative tone IP‐S resin (Nanoscribe GmbH). To work well with the Nanoscribe 3D printer, this type of IP‐S resin was customized. The Young's modulus, Poisson's ratio, and density of raw material IP‐S resin were E=4.5 GPa, v=0.43, ρ=1.11 g cm^−3^, respectively. Furthermore, the slicing and hatching distances were set equal to 1 and 0.5 µm, respectively. A drop of IP‐S resin was deposited on a ITO‐coated sodalime glass substrate with dimensions 25 mm × 25 mm × 0.7 mm and photopolymerized with a femtosecond laser operating at λ=780 nm and a 25×‐ objective. After printing, the samples were developed for 30 min in propylene glycol methy ether acetate (PGMEA) solution to remove the unexposed photoresist and rinsed for 5 min in isopropyl alcohol (IPA) to clear the developer. A laser power of 100 mW and a galvanometric scan speed of 100 mm s^−1^ were used for the whole fabrication process. In principle, when the number of cells in the metamaterials was infinite, the unit cell and the repeatable cell were equivalent. In fact, the cells of the metamaterials samples were limited. So, the repeatable cell structure in the experiments and the unit cell structure in the theoretical analysis were used. This is because the former has fewer free beam members, which can effectively reduce the boundary effect caused by the limited number of cells. To demonstrate the special mechanical properties of the auxetic metamaterials, two auxetic metamaterials with different feature parameters were designed and fabricated in this work. The first, called model A, has feature dimensions in the *x* principal direction of 2a=120 µm, the characteristic parameters $\theta_{h}$ and $\theta_{v}$ of model A are 46.4° and 45°, respectively. The dimensions of the second metamaterial, called model B, are 2a=120 µm, and $\theta_{h}=\theta_{v}=26.56^{\circ }$. The side lengths of the square section beams in both models A and B are t=6 µm. However, to make the overall dimensions of models A and B close (≈1 mm), the number of the unit cells in the three principal directions of models A and B are 4×4×8 and 5×5×14, respectively.

### Mechanical Characterization

In order to test the mechanical properties of the proposed auxetic metamaterials, they were uniaxially compressed at the constant rate of 1 µm s^−1^. At the same time, an optical microscope with a magnification of 10 was used to record the deformation process of the specimen, 1 frame per second. Then, the experimental photos were processed by MATLAB image tracking technology, as shown in Section 3 of Supplementary Information. Finally, the compressive strain was calculated using the relative displacement between points A and B, and the lateral strain was calculated using the relative displacement between points C and D. After calculating the tangent elastic constants of the metamaterial, the smooth function that comes with MATLAB was used to reduce the fluctuation of the data.

### Analytical Model

The theoretical model of the equivalent elastic constants of the metamaterial under uniaxial loading in the *z*‐direction was established in this part. Herein, it was assumed that the number of cells in the metamaterial was infinite in the three principal directions. The representative volume elements of the proposed infinite structure with applied unknown remote tensile displacement, along the *z*‐direction, as shown in Figure 5a. It is clear that this representative volume elements with periodic boundary conditions in the three principal directions due to the previous assumption. Moreover, all connection points were considered rigid to simplify analysis. In the same time, based on the geometric symmetry in three principal directions, just taking 1/8 representative volume element into consideration in this part. Figure 5b shows the equivalent boundary conditions, the displacement, the degrees of freedom, and the axial and shear force of each member of 1/8 representative volume element. In Figure 5b, $u_{i}$, $v_{i}$, $w_{i}$ represent the displacement of each endpoint in the *x*‐, *y*‐, and *z*‐directions, respectively, and $F_{\mathrm{ij}}^{N}$ and $F_{\mathrm{ij}}^{S}$ (i=A,B⋯H;j=A,B⋯H) represent the axial and shear force of each individual beam. $l_{h}$, $b_{h}$, $t_{h}$ represent the length, width, and thickness of the beam in the *x*‐*y* plane, and $l_{v}$, $b_{v}$, $t_{v}$ represent the corresponding parameters of other beams. It can be easily obtained by geometric relations that $l_{h}=a/\cos\theta_{h}$, $l_{v}=a/\cos\theta_{v}$, $b_{h}=b_{v}=t/2$, $t_{h}=t_{v}=t$.

Based on the periodicity and symmetry of the structure, endpoints that were coplanar before deformation remained coplanar after deformation, so the following relationship can be obtained, that is,

(1) $$\left\{\begin{array}{c} u_{D}=u_{G}=u_{H}\\ v_{C}=v_{F}=v_{G}\\ w_{E}=w_{F}=w_{G}=w_{H} \end{array}\right.$$

In addition, due to the similarity of AEB and CDG, the displacements of endpoints A, C, G in the *x*‐direction should satisfy the following relationship,

(2) $$u_{A}=u_{G}-u_{C}$$

Similarly, the displacements of endpoints B, D, G in *y*‐direction with the following relationship

(3) $$v_{B}=v_{G}-u_{D}$$

According to the theory of structural mechanics, the displacement of each endpoint is decomposed along three principal directions, and the axial force of each beam can be calculated as follows,

(4) $$F_{\mathrm{ij}}^{N}=\frac{Eb_{s}t_{s}}{l_{s}}\left(u_{i}k+v_{i}k+w_{i}k+u_{j}k+v_{j}k+w_{j}k\right)$$

On account of the first‐order shear deformation beam theory and using the coordinate transformation method,^[^ ^14^ ^]^ the shear force of each beam can be calculated as follows,

(5) $$F_{\mathrm{ij}}^{S}=\frac{u_{i}k+v_{i}k+w_{i}k+u_{j}k+v_{j}k+w_{j}k}{\frac{l_{s}^{3}}{Eb_{s}t_{s}^{3}}+\frac{6l_{s}}{5Gb_{s}t_{s}}}$$

where i=A,B⋯H, j=A,B⋯H, $k=0,\;\pm \sin\theta_{s},\;\pm \cos\theta_{s}$, s=h or *v*, *E* and *G* are the Young's modulus and shear modulus of the raw material, respectively. The specific expressions of the axial and shear forces for each beam are presented in the first section of the Supplementary Information.

Under quasi‐static loading, each end point always satisfies the force equilibrium condition. Considering the *x*‐axial force balance of point A, the following equation can be obtained

(6) $$\begin{array}{ccc} & & F_{\mathrm{AB}}^{N}\cos\theta_{h}-F_{\mathrm{AB}}^{S}\sin\theta_{h}-F_{\mathrm{AD}}^{N}\sin\theta_{h}-F_{\mathrm{AD}}^{S}\cos\theta_{h}\\ & & \;+\;F_{\mathrm{AE}}^{N}\cos\theta_{v}-F_{\mathrm{AE}}^{S}\sin\theta_{v}=0 \end{array}$$

Similarly, considering the *y*‐axial force balance of point B, the following equation can be obtained

(7) $$\begin{array}{ccc} & & F_{\mathrm{AB}}^{N}\sin\theta_{h}+F_{\mathrm{AB}}^{S}\cos\theta_{h}-F_{\mathrm{BC}}^{N}\cos\theta_{h}+F_{\mathrm{BC}}^{S}\sin\theta_{h}-F_{\mathrm{BF}}^{N}\cos\theta_{v}\\ & & \;+\;F_{\mathrm{BF}}^{S}\sin\theta_{v}=0 \end{array}$$

Moreover, the resultant force along the *y*‐axis on the right boundary of the unit cell must be zero,

(8) $$\begin{array}{ccc} & & F_{\mathrm{BF}}^{N}\cos\theta_{v}-F_{\mathrm{BF}}^{S}\sin\theta_{v}+F_{\mathrm{BC}}^{N}\cos\theta_{h}-F_{\mathrm{BC}}^{S}\sin\theta_{h}+F_{\mathrm{CD}}^{N}\sin\theta_{h}\\ & & \;+\;F_{\mathrm{CD}}^{S}\cos\theta_{h}=0 \end{array}$$

Analogously, the *x*‐axial resultant force on the front boundary of the unit cell also should be zero, i.e.

(9) $$\begin{array}{ccc} & & F_{\mathrm{AE}}^{N}\cos\theta_{v}-F_{\mathrm{AE}}^{S}\sin\theta_{v}+F_{\mathrm{AD}}^{N}\sin\theta_{h}+F_{\mathrm{AD}}^{S}\cos\theta_{h}\\ & & \;+\;F_{\mathrm{CD}}^{N}\cos\theta_{h}-F_{\mathrm{CD}}^{S}\sin\theta_{h}=0 \end{array}$$

Here, it is assumed that the structure is subjected to displacement loads in the *z*‐direction, i.e. $w_{E}$ is known. By combining Equations (1), (2), (3), (4), (5), (6), (7), (8), (9), the displacements of each endpoint of the studied structure in the three principal directions can be obtained.

Therefore, the effective strain in the *x*‐ and *z*‐directions and effective Poisson's ratio of the structure can be calculated

(10) $$\left\{\begin{array}{c} \varepsilon_{x}=u_{D}/\left(l_{h}\sin\theta_{h}+l_{h}\cos\theta_{h}+2b_{v}\right)\\ \varepsilon_{z}=w_{E}/\left(l_{v}\sin\theta_{v}+b_{h}\right)\\ \nu_{\mathrm{zx}}=-\varepsilon_{x}/\varepsilon_{z} \end{array}\right.$$

The relationship between the equivalent stress in the *z*‐directions $\sigma_{z}$ and $w_{E}$ can be expressed as

(11) $$\begin{array}{ccc} & & 2\left(F_{\mathrm{AE}}^{N}\sin\theta_{v}+F_{\mathrm{AE}}^{S}\cos\theta_{v}+F_{\mathrm{BF}}^{N}\sin\theta_{v}+F_{\mathrm{BF}}^{S}\cos\theta_{v}\right)\\ & & \;=\sigma_{z}{\left(l_{h}\sin\theta_{h}+l_{h}\cos\theta_{h}+2b_{v}\right)}^{2} \end{array}$$

The expression of $\sigma_{z}$ can be obtained by Equation 11. Then, the equivalent Young's modulus of the structure is

(12) $$E_{z}=\frac{\sigma_{z}}{\varepsilon_{z}}$$

### Finite Element Simulation

Commercial finite element analysis software ABAQUS/Explicit (version 2021) was used to get the mechanical response of the auxetic structures under uniaxial compression loading. The models with the same number of unit cells as the specimens for experiments were established, which consists of the top plate, specimen, and bottom platen. The unit cells were meshed via hexahedral solid elements C3D8R with approximate of 2.5 µm. During the whole simulation, all plates were simulated using a discrete rigid and coupled all degrees of freedom with a reference point located at its volume center. The reference point of the top plate was subjected to a displacement load along the *z‐*direction, and the other five degrees of freedom of this reference point were fixed. In contrast, all degrees of freedom of the reference point of the bottom plate were fully fixed. Moreover, general contact interaction was established between the rigid plates and the element‐based surfaces of the structure.

## Conflict of Interest

The authors declare no conflict of interest.

## Author Contributions

L.W. conceived the study and carried out the experimental testing, theoretical and computational analysis. B.W. and V.L. supervised and M.K. and G.U. prepared samples with 3D printing. J.A.I.M. and K.K.D. carried out theoretical analysis and used MATLAB to process experimental results. All authors worked together to write and revise the manuscript.

## Supporting information

Supporting Information

Supplemental Video 1

## Data Availability

The data that support the findings of this study are available in the supplementary material of this article.
