# Supplementary material for: 3D Auxetic Metamaterials with Elastically‐Stable Continuous Phase Transition
Source: Adv Sci (Weinh). 2022 Oct 18;9(34):2204721. doi: 10.1002/advs.202204721 (PMC9731712; doi:10.1002/advs.202204721)
Supplement: Supplementary file 1 — Supporting Information [file ADVS-9-2204721-s001.pdf]

Supplementary information for

Three-dimensional Auxetic Metamaterials with Elastically-stable Continuous Phase Transition

Lianchao Wang[1,2], Gwenn Ulliac[2], Bing Wang[1,*], Julio A. Iglesias Martínez[2], Krzysztof K. Dudek[2,3], Vincent Laude[2], Muamer Kadic[2,*]

[1]National Key Laboratory of Science and Technology on Advanced Composites in Special Environments, Harbin Institute of Technology, Harbin, 150001, PR China

[2] Institut FEMTO-ST, CNRS UMR 6174, University Bourgogne Franche-Comté, Besançon, 25000, France

[3] Institute of Physics, University of Zielona Gora, ul. Szafrana 4a, 65-069 Zielona Gora, Poland

[*]Corresponding Author:

Prof. Bing Wang,

Email Address:wangbing86@hit.edu.cn

Prof. Muamer Kadic,

Email Address:muamer.kadic@gmail.com

1. Analytical Model

1.1 Axial force and shear fore of each beam

According to the theory of structural mechanics, the displacement at each endpoint is decomposed along three principal directions, and the axial force on each beam can be calculated as follows,

$$\begin{cases} F_{AB}^{N} = \dfrac{Eb_h t_h}{l_h}\left(u_A \cos\theta_h + v_B \sin\theta_h\right) \\ F_{AD}^{N} = \dfrac{Eb_h t_h}{l_h}\left(-u_A \sin\theta_h + u_D \sin\theta_h + v_D \cos\theta_h\right) \\ F_{BC}^{N} = \dfrac{Eb_h t_h}{l_h}\left(-v_B \cos\theta_h + u_C \sin\theta_h + v_C \cos\theta_h\right) \\ F_{CD}^{N} = \dfrac{Eb_h t_h}{l_h}\left(u_D \cos\theta_h - v_D \sin\theta_h - u_C \cos\theta_h + v_C \sin\theta_h\right) \\ F_{AE}^{N} = \dfrac{Eb_v t_v}{l_v}\left(u_A \cos\theta_v + w_E \sin\theta_v\right) \\ F_{BF}^{N} = \dfrac{Eb_v t_v}{l_v}\left(-v_B \cos\theta_v + v_F \cos\theta_v + w_F \sin\theta_v\right) \end{cases}$$

On account of first-order shear deformation beam theory and using the coordinate transformation method[1], the shear force on each beam can be calculated as follows,

$$\begin{cases} F_{AB}^{S} = \dfrac{-u_A \sin\theta_h + v_B \cos\theta_h}{\dfrac{l_h^3}{Eb_h t_h^3} + \dfrac{6l_h}{5Gb_h t_h}} \\ F_{AD}^{S} = \dfrac{-u_A \cos\theta_h + u_D \cos\theta_h - v_D \sin\theta_h}{\dfrac{l_h^3}{Eb_h t_h^3} + \dfrac{6l_h}{5Gb_h t_h}} \\ F_{BC}^{S} = \dfrac{v_B \sin\theta_h + u_C \cos\theta_h - v_C \sin\theta_h}{\dfrac{l_h^3}{Eb_h t_h^3} + \dfrac{6l_h}{5Gb_h t_h}} \end{cases}$$

$$
\begin{cases}
F_{CD}^{S} = \dfrac{-u_D \sin\theta_h - v_D \cos\theta_h + u_C \sin\theta_h + v_C \cos\theta_h}{\dfrac{l_h^{\ 3}}{Eb_h t_h^{\ 3}} + \dfrac{6l_h}{5Gb_h t_h}} \\
F_{AE}^{S} = \dfrac{-u_A \sin\theta_v + w_E \cos\theta_v}{\dfrac{l_v^{\ 3}}{Eb_v t_v^{\ 3}} + \dfrac{6l_v}{5Gb_v t_v}} \\
F_{BF}^{S} = \dfrac{v_B \sin\theta_v - v_F \sin\theta_v + w_F \cos\theta_v}{\dfrac{l_v^{\ 3}}{Eb_v t_v^{\ 3}} + \dfrac{6l_v}{5Gb_v t_v}}
\end{cases}
$$

where E and G are the Young's modulus and the shear modulus of the raw material, respectively.

1.2 Comparison of the results of equivalent elastic constants obtained by three methods

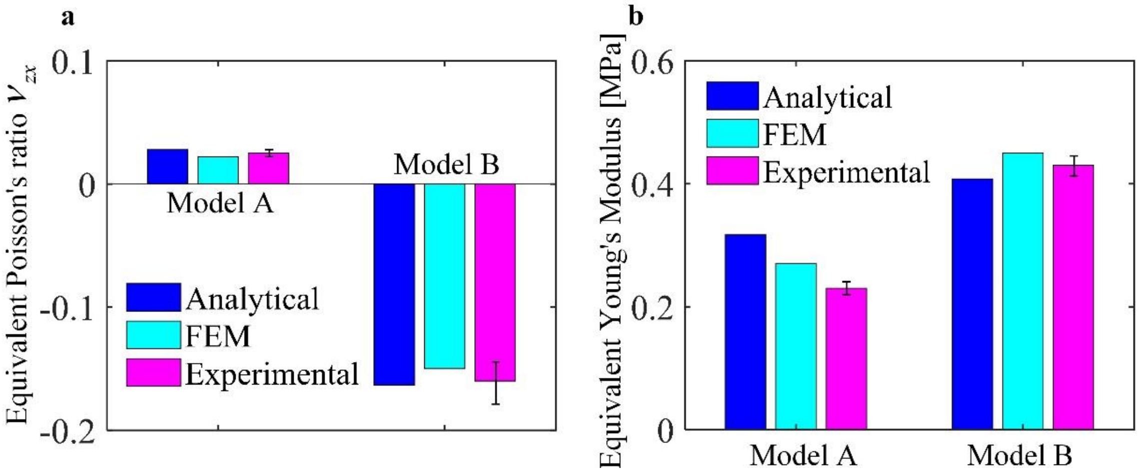

Figure S1: Comparison of equivalent elastic constants obtained by three methods: (a) Poisson's ratio and (b) Young's modulus.

The equivalent elastic constants of OBCC mechanical metamaterials obtained by the analytic model, the finite element method (FEM) and experiment are shown in Fig. S2. Overall, the equivalent Poisson's ratio and the equivalent Young's modulus for models A and B match well. This proves the correctness of the theoretical model established in this paper and the rationality of the finite element model. Of course, there are some slight

discrepancies. The difference between the theoretical and simulated results may be due to different underlying assumptions. For example, the theoretical model assumes periodic boundaries with an infinite number of structural elements, whereas the number of elements in a finite element model is limited, i.e. boundary effects can affect the finite element results. The discrepancies between experimental results and FEM results may be caused by errors during the preparation of the specimen or by errors during the test. In conclusion, the results obtained by these three methods are consistent.

2. Evaluation of the Energy Absorption Ability of Auxetic Metamaterials

In principle, due to the deformation characteristics of compression-shrinkage, most auxetics have great potential application value for energy absorption. Most reported negative Poisson's ratio metamaterials, however, have weak energy absorption ability since they are bending-dominated structural materials. Therefore, auxetic tensile-dominated metamaterials are expected to show better energy absorption. The main purpose of this section is to evaluate the energy absorption ability of metamaterials to explore their potential application to energy absorption.

The strain versus stress curves for models A and B under large deformation are shown in Figure S3 a-b. It can be seen that the energy absorption curves of models A and B have similar shapes. Some slight differences remain, however. First, the energy absorption curve of model A shows obvious fluctuations, that are caused by the instability in the vertical direction of the V-shaped beam of the unit cell, under large deformation. The energy absorption curve of model B is relatively smooth in comparison. The energy absorption curve of model B also shows peaks and troughs, but it is obvious that the peaks and troughs

of model A are sharper. This is because the height of the V-shaped beam in the unit cell of model A is larger, i.e. the value of θ_v is larger (θ_v is 45^0 for model A and 26.56^0 for model B).

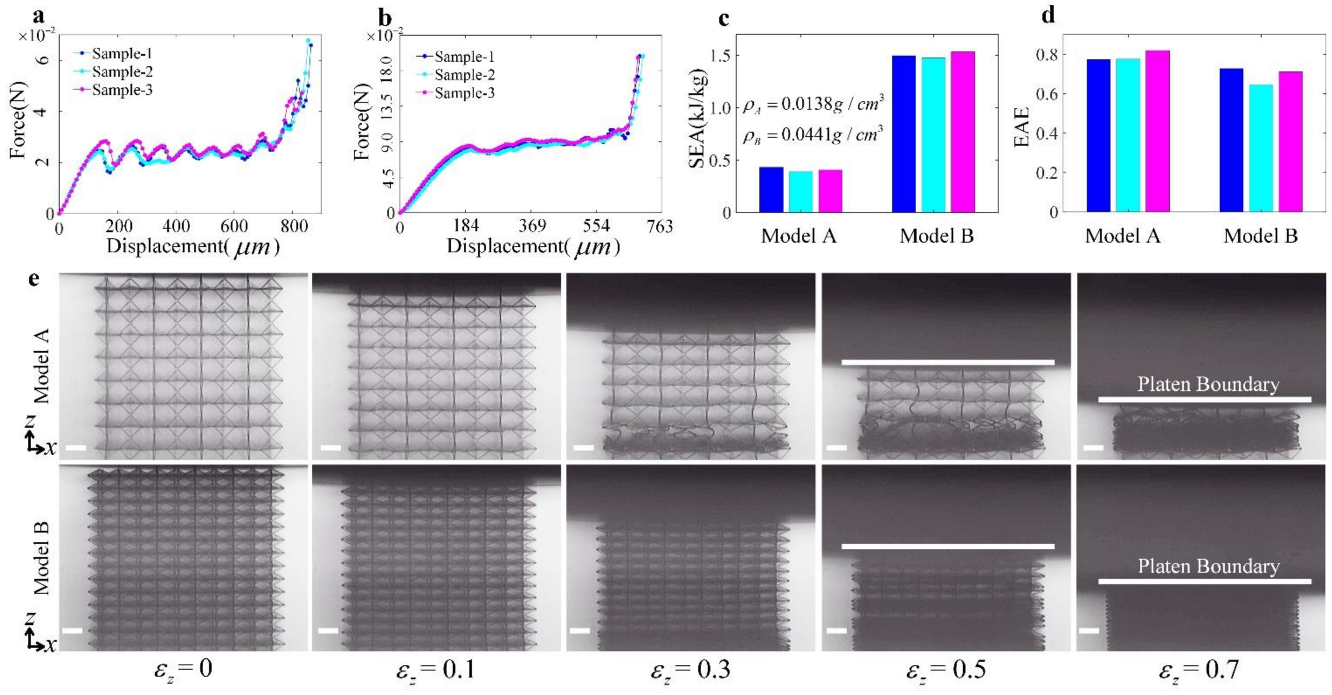

Figure S2: Evaluation of specific energy absorption and energy absorption efficiency of models A and B. (a)-(b) Compressive displacement versus force curves of models A and B under large deformation. For all curves, the number of peaks is equal to the number of layers of the metamaterials. (c)-(d) Specific energy absorption (SEA) and energy absorption efficiency (EAE) of models A and B, respectively. (e) Experimental results of deformation rules of models A and B during the large compression process. The scale bar length is 100 μm.

It is worth noting here that the number of peaks in the energy absorption curves is equal to the number of layers of the metamaterial unit cell, since each peak corresponds to the instability of one given layer. Moreover, because the dimensions of model B in the *x*- and *y*-directions are smaller (i.e. the value of $a(1+\tan\theta_h)$ is smaller), model B has larger stiffness, strength, and energy absorption capacity than model A. A smaller value for $a(1+\tan\theta_h)$ results in a stronger constraint on the V-shaped beam in the vertical direction, so that the V-shaped beam is less prone to deformation. The lateral deformation of models A and B are

shown in Figure S3e. It is clear that both models A and B fail regularly layer by layer until they are fully compacted. Moreover, they remain contracted throughout the compression process (until the compressive strain exceeds 70%), which indicates the metamaterial retains a negative Poisson's ratio during the whole process. In addition, it also has the same deformation characteristics in the y direction due to the structure of the auxetic metamaterial in the x and y directions being identical. Supplementary Videos show the auxetic metamaterial compressive deformation observed from different directions.

Specific energy absorption (SEA) and energy absorption efficiency (EAE) are important parameters for evaluating the performance of energy-absorbing materials. The mass of the auxetic metamaterial m can be calculated by $m = 8N(2\sqrt{a^2+b^2} + 2\sqrt{a^2+c^2} - 5t)t^2\rho$, where N is the number of unit cells and $\rho = 1.11\,\mathrm{g/cm^3}$ is the density of the raw material IP-S. Herein, the specific energy absorption and energy absorption efficiency of both models A and B have been calculated and are shown in Figure S3 c-d. It can be seen that models A and B have different specific energy absorptions, $0.47\,\mathrm{kJ/kg}$ and $1.5\,\mathrm{kJ/kg}$, respectively. More importantly, models A and B have relatively low densities, $0.01238\,\mathrm{g/cm^3}$ and $0.0441\,\mathrm{g/cm^3}$, respectively. For such low-density auxetic metamaterials, they exhibit excellent energy absorption capacity, as explained by two main reasons. First, the raw material, i.e. the custom IP-S resin, has outstanding specific mechanical properties (large elastic modulus and low density). Second, the proposed metamaterials are structurally efficient due to the deformation mode being tension/compression-dominated, whereas most other auxetics are bending-dominated. Moreover, the low density broadens the effective strain range of the metamaterials, especially for model A, that is still not fully dense even though the compressive strain exceeds 0.7. In addition, both metamaterials have large energy absorption

efficiencies of 0.78 and 0.72, respectively. Overall, the low density (light mass), the regular failure mode, the high specific energy absorption, and the energy absorption efficiency indicate that the designed auxetic metamaterials could find potential applications in the field of energy absorption, especially for advanced sports equipment and aerospace applications.

3. Matlab processing of experimental results

The experimental results are processed using image tracking implemented in MATLAB, as illustrated in Figure S3. RGB values of the picture are first converted to grayscale values and then tracked according to the intensity of each pixel. Pixels that can be identified and tracked throughout the compression process are shown as green dots in Figure S3. Here, we select the trajectories of the four points A, B, C, and D to calculate the equivalent Poisson's ratio of the structure. Strains are evaluated as follows

$$\varepsilon_x = \frac{\Delta_{C\_x} - \Delta_{D\_x}}{L_{CD}}, \varepsilon_z = \frac{\Delta_{A\_z} - \Delta_{B\_z}}{L_{AB}}$$

where Δ_{C_x} and Δ_{D_x}B represent the displacement of points C and D in the *x*-direction， Δ_{A_z} and Δ_{B_z} represent the displacement of points A and B in the *z*-direction. L_{AB} and L_{CD} represent the initial distance between points A and B, and points C and D.

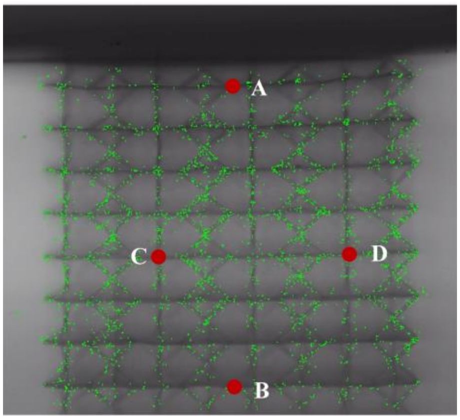

Figure S3: Illustration of image processing using MATLAB to evaluate the equivalent Poisson's ratio from reference points selected inside the experimental observation of the deformed sample.

References

[1] Gao, Y., Wei, X., Han, X., Zhou, Z. & Xiong, J. Novel 3d auxetic lattice structures developed based on the rotating rigid mechanism. Int. J. Solids Struct. 233, 111232 (2021).

[2] K. Bertoldi, P. M. Reis, S. Willshaw, T. Mullin. Negative Poisson’s Ratio Behavior Induced by an Elastic Instability. Advanced materials, 22, 3 361(2010).
